# Clinical and Family Implications of Cannabidiol (CBD)-Dominant Full-Spectrum Phytocannabinoid Extract in Children and Adolescents with Moderate to Severe Non-Syndromic Autism Spectrum Disorder (ASD): An Observational Study on Neurobehavioral Management

**DOI:** 10.3390/ph17060686

**Published:** 2024-05-27

**Authors:** Jeanne Alves de Souza Mazza, Lisiane Seguti Ferreira, Alice de Faria Martins-Vieira, Doris Day Lopes Beserra, Victor Alves Rodrigues, Renato Malcher-Lopes, Fabio V. Caixeta

**Affiliations:** 1 University Hospital of Brasilia, Campus Darcy Ribeiro, Brasilia 70840-901, Brazil; jeannemazzamed@gmail.com (J.A.d.S.M.); lisianeseguti@gmail.com (L.S.F.); dorislopesb@gmail.com (D.D.L.B.); victoralvesrodrigues2017@gmail.com (V.A.R.); 2 Department of Physiological Sciences, University of Brasilia, Campus Darcy Ribeiro, Brasilia 70910-900, Brazil; alice.faria.martins@gmail.com (A.d.F.M.-V.); malcherlopes@gmail.com (R.M.-L.)

**Keywords:** idiopathic autism, cannabinoid treatment, cannabis, pediatric neurodevelopmental disorders

## Abstract

Autism Spectrum Disorder (ASD) encompasses a wide range of neurodevelopmental conditions characterized by deficits in social interaction, communication and behavior. Current pharmacological options are limited and feature significant side effects. In this study, we conducted a retrospective, observational, and cross-sectional cohort study to evaluate the effects of Cannabidiol (CBD)-dominant, full-spectrum cannabis extract, containing Tetrahydrocannabinol (THC) in a ratio of 33:1 (CBD:THC), on non-syndromic children and adolescents (5–18 years old) with moderate to severe ASD. Thirty volunteers were recruited, underwent neuropsychological evaluations and were treated with individualized doses of CBD-dominant extract. Clinical assessments were conducted by the designated clinician. Additionally, parents or caregivers were independently interviewed to assess perceived treatment effects. We found significant improvements in various symptomatic and non-symptomatic aspects of ASD, with minimal untoward effects, as reported by both clinical assessments and parental perceptions. The observed improvements included increased communicative skills, attention, learning, eye contact, diminished aggression and irritability, and an overall increase in both the patient’s and family’s quality of life. Despite its limitations, our findings suggest that treatment with full-spectrum CBD-dominant extract may be a safe and effective option for core and comorbid symptoms of ASD, and it may also increase overall quality of life for individuals with ASD and their families.

## 1. Introduction

Autism Spectrum Disorder (ASD) comprises a diverse group of neurodevelopmental conditions that encompass deficits in the domains of social interaction, communication, and behavior [1]. According to the Diagnostic and Statistical Manual of Mental Disorders 5th Edition, Text Revision, the criteria for diagnosing ASD are subdivided into two categories. The first refers to persistent deficits in social communication and social interaction in multiple contexts, and the second refers to restrictive patterns and repetitive behaviors, interests or activities. The spectrum in which individuals with ASD are inserted is characterized by a set of behaviors that can be clinically presented in different ways, with different associated characteristics [2]. When determining the severity of ASD, the level of support needed in the social and behavioral domains is taken into account, indicating (level 1) the need for some support, (level 2) substantial support or (level 3) very substantial support [1]. This condition affects not only individuals with ASD but also their families and caregivers, since autism symptoms often interfere with the patient’s autonomy and social development, which can make everyday life stressful for everyone involved. As research data have accumulated and our understanding of ASD has improved, individuals diagnosed with this condition have garnered increased recognition and visibility within society; however, significant challenges persist in overcoming the stigma associated with autism [3].

In recent decades, great advances have been made in the field of development in regard to genetic, biologic and environmental factors related to ASD. However, some etiological aspects and underlying mechanisms associated with autistic symptoms remain poorly understood [4]. The causes behind these disorders are multifactorial, and individuals with ASD often present comorbidities that vary in their etiologies and severities [5]. Some hypotheses have been raised to try to explain, from a neurophysiological perspective, the functional differences presented by people diagnosed with ASD. The hypothesis of neuronal hyperexcitability stands out [6], in accordance with the higher incidence of epilepsy in autistic patients, and there is evidence of epileptiform alterations even in individuals with ASD without an associated diagnosis of epilepsy [7]. In the same sense, the intense world hypothesis also associates ASD with excessive neuronal activity and connectivity [8]. Studies with family members have indicated that there is a genetic basis that considerably impacts susceptibility to ASD development, and genetic sequencing has been crucial in the quest to elucidate the genetic architecture behind these disorders [9]. Moreover, the etiopathology seems to be, among other factors, associated with genetic or epigenetic changes related to neuronal functioning. More specifically, many findings indicate that the expression of endogenous cannabinoids and endocannabinoid receptors is altered in individuals with ASD [10]. Also, a correlation between the endocannabinoid system and the autistic phenotype has already been described due to the involvement of endogenous cannabinoids in the control of emotional and behavioral responses and in the reactivity to context and social interactions [11]. In fact, it has recently been observed that the plasmatic concentration of one of the main endocannabinoids, Anandamide, is reduced in children on the spectrum [12].

Behavioral therapy coupled with pharmacological interventions typically serves as the primary course of treatment for ASD. In spite of continuous improvements to these approaches, it is reported that around 40% of pediatric patients diagnosed with ASD undergoing treatment continue with maladaptive behaviors [13]. Despite recent advances, the range of treatment options available for the neuropsychiatric symptoms of autism is still considerably restricted, and currently available pharmacological alternatives are not specific for core ASD symptoms. In the United States, for example, only two medications, Risperidone and Aripiprazole, have been approved specifically for the treatment of children with ASD, and both drugs are primarily antipsychotics. Antipsychotics, as well as antidepressants and anxiolytics, can be useful and efficient in treating and controlling certain symptoms [14], such as controlling self- and hetero-aggressiveness [15], while antiseizure medication, in addition to controlling seizures, can be, secondarily, beneficial for sleep quality and other specific behavioral aspects. On the other hand, antipsychotics are known to cause important side effects, such as endocrine and metabolic dysregulations, as well as cardiac changes and extrapyramidal effects [16]. The intrinsic heterogeneity of this diagnosis also impacts the outcome of treatment, since the origin and presentation of symptoms differ considerably from patient to patient. Furthermore, new symptoms of ASD may appear throughout the individual’s life, while other symptoms may naturally reduce in severity, which must be taken into account when choosing the psychopharmacological intervention to be adopted [17]. Additionally, none of the conventional treatments used for behavioral management and treatment of people with ASD have shown significant benefits in regard to social interaction, communication, motor function and intellectual development [17,18], which constitute a large part of the dysfunctional core that impacts the daily lives of individuals with ASD and their families.

Given these limitations, there is an ongoing search for novel treatment alternatives for ASD. Extracts from *Cannabis sativa*, containing phytocannabinoids such as CBD and THC, have gained great interest recently. Phytocannabinoids are being explored for treating various disorders and diseases, such as epilepsy, anxiety, chronic pain, spasticity, Parkinson’s disease, psychosis, aggression, attention deficit hyperactivity disorder (ADHD) and ASD [19,20]. While some studies have identified methodological limitations, such as small and heterogeneous cohorts, study design and lack of standardized doses [20], there is substantial evidence supporting the efficacy of phytocannabinoids in treating specific disorders. For instance, CBD’s role in managing seizures has been extensively studied, with many randomized trials demonstrating significant reductions or even cessation of seizures in a significant portion of cases [19,20]. Ongoing clinical use of CBD has further bolstered its reputation as a safe and effective alternative for individuals with epilepsy, with increasing demand from both children and adults with treatment-resistant seizures [19].

In the realm of ASD, both preclinical and clinical studies indicate CBD’s potential in improving social behavior and addressing comorbidities like sleep disorders, ADHD, anxiety and seizures [19]. However, evidence supporting CBD’s effectiveness in treating compulsive behavior, mood disorders, cognitive impairments and aggression remains limited. Reported side effects include drowsiness, gastrointestinal issues, fatigue, vomiting, lethargy, changes in appetite and insomnia [20,21,22]. Moreover, there is currently a scarce amount of data regarding CBD’s interactions with other medications [21].

In conclusion, treatment with phytocannabinoids has shown promise in ameliorating several core autistic symptoms, as well as many comorbid symptoms, with mild and infrequent side effects compared to conventional treatments [23]. Prospective and retrospective studies presented promising results, demonstrating the therapeutic efficacy and safety of using pure CBD or CBD-rich extracts for this population, positively impacting both individuals with ASD and their families’ quality of life [22,24,25,26,27,28,29]. Here we present a retrospective, observational and cross-sectional cohort study in which we evaluate the effects of full-spectrum CBD oil (containing THC in a ratio of 33:1) and its potential therapeutic value for non-syndromic children and adolescents on the autism spectrum.

## 2. Materials and Methods

Thirty volunteers, featuring 24 males aged between 5 and 18 years (mean 11.2 years) diagnosed with moderate to severe ASD (Table 1), were recruited at the University Hospital of the University of Brasília (HUB—UnB), Brazil. The University Hospital Research Ethics Committee and the National Committee of Ethics in Research (CEP-CONEP) approved the study (CAAE# 34383220.9.0000.5558), and all subjects provided written informed consent before participation. All volunteers were patients at the HUB undergoing regular medical supervision before CBD oil treatment onset, and they had already received at least one type of drug treatment previously, such as antipsychotics, melatonin, antiseizure medication, antidepressants or anxiolytics.

At the beginning of treatment, all volunteers went through neuropsychological assessment, with the Wechsler Scale (WISC-IV or WASI) being used for volunteers over six years old and the SON-R Scale being used for non-verbal volunteers or those under six years old. Out of the 30 volunteers, 28 had cognitive impairments, being borderline or intellectually disabled (Table 1). In order to obtain as homogeneous a sample as possible, volunteers with a diagnosis or suspicion of genetic or neuro-metabolic syndromes or diseases, as well as epilepsy, were not included. In addition, the sample exclusion criteria also included the presence of serious organic diseases, such as heart disease, liver disease and nephropathy, and somatic neurological and neuroimaging examinations indicative of disorders other than ASD, such that only volunteers with non-syndromic (or idiopathic) ASD were recruited [30].

All participants were treated with full-spectrum cannabidiol (CBD) extract containing THC at a ratio of 33:1 (see Section 2.1). The average treatment duration was 6.6 months. Two volunteers chose to discontinue the treatment due to untoward effects before 6 months (25m and 28f) but nevertheless completed all assessment and interviews like the other participants.

The initial dose for all individuals was 1 mg/kg/day of CBD (0.03 mg/kg/day of THC), from which an individualized and progressive titration regimen was adopted, according to patient responses, similar to the scheme used in [31]. In this process, there was a gradual increase in doses (unless dose increase led to the appearance of side effects or worsened any behavioral aspect), reaching final average doses of 3.11 mg/kg/day of CBD (0.09 mg/kg/day of THC). This final dose is compatible with the effective dose for this population, as estimated by previous studies [27,29,32,33]. All these data are summarized in Table 1. Other medication that volunteers received at the onset and at the end of treatment, as well as eventual untoward effects, are listed in Table 7, in Section 3.3.

### 2.1. Acquisition of Full-Spectrum CBD Extract

The cannabidiol extract prescribed to volunteers, Nabix 10.000, with a composition of 100 mg/mL of CBD and 3 mg/mL of THC (or 33:1 CBD-THC), was donated by the manufacturer, FarmaUSA. The company did not have any participation in the design, nor analysis, nor writing of this manuscript, nor did it provide any benefit or remuneration to the authors, acting solely as the donor of the medicines used.

### 2.2. Data Acquisition and Analysis

#### 2.2.1. Clinical Assessment

In order to follow up the evolution of social interaction, communication and behavior, the pediatric neurologist responsible for the patients (J.A.S.M.) recorded various aspects using a questionnaire based on the DSM-V diagnostic criteria A and B for ASD (F84), filled out before the start of treatment and after 1, 3 and 6 months of treatment of each patient. The following aspects related to adaptive behavior were recorded: 1. Eye contact (EC), 2. Attention to Others (AO), 3. Company seeking (CS), 4. Affectivity (AF), 5. Communicative Intent (CI), 6. Expressive Language (EL), 7. Receptive Language (RL), 8. Learning (LE), 9. Dysfunctional/Repetitive Play (DRP) and 10. Daily Life Activities (DLA). Restricted, repetitive patterns of behavior, interests, or activities recorded included 1. Self-aggressiveness (S-AGG), 2. Aggressiveness towards others (AGG), 3. Irritability (IRR), 4. Psychomotor Agitation (PA), 5. Motor Stereotypies (MST), 6. Vocal Stereotypies (VST), 7. Echolalias (ECH), 8. Ritualistic Behaviors (RB), 9. Sensory Dysfunction (SD), 10. Sleep Problems (SP), 11. Excessive appetite (EA) and 12. Avoidance and Restriction of Food Intake (ARFI). In addition, volunteers and caregivers’ quality of life during the study were also assessed. The results of the final clinical assessment after 6 months of treatment (change in relation to the initial condition, before treatment onset) were used for statistical analysis.

#### 2.2.2. Parent/Caregiver-Reported Outcome Survey

At the end of the treatment, semi-structured interviews were carried out by 2 interviewers (A.F.M-V. and F.V.C) who did not participate and were not involved in the course of the patient’s treatment. The interviews happened in person or over the phone with either a parent or the main caregiver of each participant present in order to assess the family’s perception about the effects of the treatment through a “Parent/Caregiver-reported outcomes survey”, as previously described in [31]. The goal of the interviews was to evaluate the impression of people who spent more time with the patient in relation to their autistic symptoms as well as to evaluate the quality of life of both the patient and their family.

The interview was filled out in an online form divided into 12 sections corresponding to different symptomatic or non-symptomatic categories, as described in [31]. The categories corresponded to the following: 1. Attention Deficit and Hyperactivity (ADH), 2. Abnormal Behaviors (AB), 3. Sadness, Melancholy and Bad Mood (SMBM), 4. Motor Difficulties (MD), 5. Dependence for Daily Activities (DDA), 6. Communication and Personal Interaction (CPI), 7. Cognitive Difficulties (CD), 8. Sleep Problems (SP) and 9. Avoidance or Restriction of Food Intake (ARFI).Non-symptomatic categories assessed were: 10. Positive Mood States (PM), 11. Patient’s Quality of Life (PQoL) and 12. Family’s Quality of Life (FQoL).

Each section contained multiple-choice questions to measure the perceived treatment effect for each aspect evaluated. The response options corresponded to a 5-level Likert-type scale, in addition to the “Not Applicable” option, when the symptom was not present, as previously described in Montagner et al., 2023. More specifically, parents were explicitly asked what they thought was the effect of the treatment on the aspect in question and could choose one of the following answers: “Not Applicable”, “There was great worsening”, “There was moderate worsening”, “There were no changes”, “There was moderate improvement” and “There was great improvement”. In other words, for volunteers who presented the type of symptom in question, the respondent had to choose one of the five options on the scale used.

Moreover, within the same form, unstructured responses to open-ended questions were solicited wherein participants provided detailed narratives regarding the observed alterations attributed to the intervention for each respective category. This approach aimed to ascertain the precision of participants in discerning the specific symptoms under discussion in addition to supporting research with additional information on practical and subjective aspects not covered by the multiple-choice questions.

For data compilation, the 5 possible answers about the effects of treatment in a given category were translated numerically into values, with −2 and −1 meaning great and moderate worsening, respectively, 0 meaning unchanged symptom severity and +2 and +1 meaning great and moderate improvement, respectively. For volunteers who did not present the symptom in question, the nomenclature “NaN” was used. In the tables, the individual results of each patient are presented for each category evaluated, in which cases of improvement, worsening or no change are presented, in their corresponding numerical values, as well as cases in which there was no manifestation of the symptom (Not a Number, or NaN). For clarity, in the figures, we standardized any value that was marked as NaN in the tables to zero. That is, if a participant did not present the symptom of a given category either at the beginning or at the end of treatment, this was compiled as “0” or without a change in the figures. Compiled data were plotted into figures using MATLAB R2023b using the “barh” function, graphed as in daydreamingnumbers.com/blog/4-ways-to-visualize-likert-scales (accessed on 2 May 2024).

## 3. Results

### 3.1. Results by Symptom Category

#### 3.1.1. Clinical Assessment

**Figure 1:**
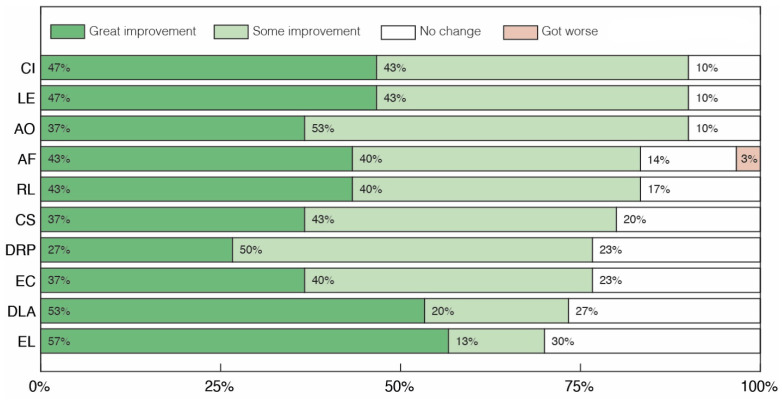
Clinical assessment of changes in social cognition after treatment with cannabidiol extract. Communicative Intent (CI), Learning (LE), Attention to Others (AO), Affectivity (AF), Receptive Language (RL), Company seeking (CS), Dysfunctional/Repetitive Play (DRP), Eye Contact (EC), Daily Life Activities (DLA) and Expressive Language (EL).

**Figure 2:**
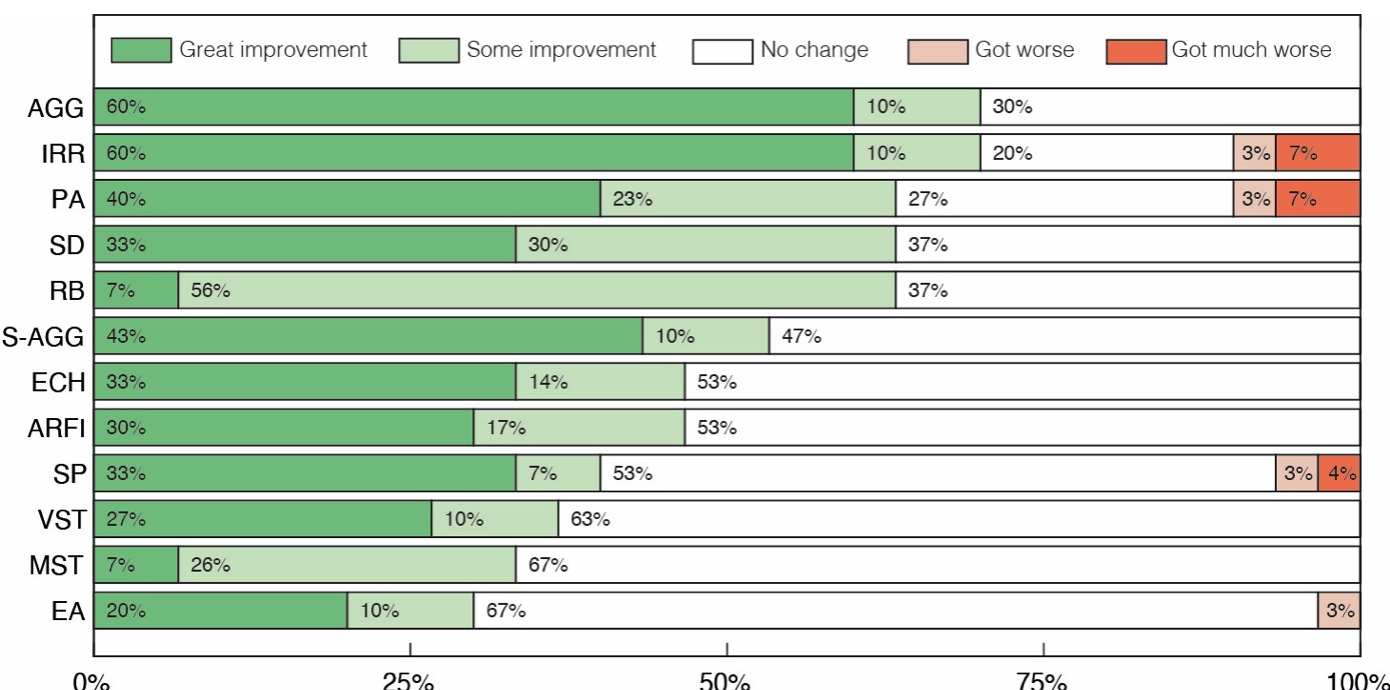
Clinical assessment of changes in repetitive/restrictive and dysfunctional behavior after treatment with cannabidiol extract. Aggressiveness towards others (AGG), Irritability (IRR), Psychomotor Agitation (PA), Sensory Dysfunction (SD), Ritualistic Behaviors (RB), Self-aggressiveness (S-AGG), Echolalias (ECH), Avoidance and Restriction of Food Intake (ARFI), Sleep Problems (SP), Vocal Stereotypies (VST), Motor Stereotypies (MST) and Excessive Appetite (EA).

Clinical assessment of our cohort is presented in Figure 1 and Figure 2. Overall, volunteers presented more positive effects on Social Cognition than on Repetitive/Restrictive and Dysfunctional Patterns. At least 70% of volunteers showed some level of improvement (great improvement + moderate improvement) in all categories evaluated in the first set of criteria (Figure 1). In particular, the categories with the highest percentage of volunteers with moderate to great improvement were Communicative Intent (CI), Learning (LE) and Attention to others (AO), while the categories with the highest percentage of reports of great improvement were Expressive Language (EL) and Daily Life Activities (DLA). It is also worth mentioning that, although some participants showed increased weight at the end of the study, there was almost no change in Body Mass Index (BMI) for participants, with an average change of −0.35 (Supplementary Table S1).

Analyzing the results for the second group of clinical aspects (Figure 2), improvements greater than or equal to 53% were reported in 6 of the 12 categories. In particular, in the Aggressiveness towards others (AGG) and Irritability (IRR) categories, 70% of volunteers achieved some level of improvement, while in the Psychomotor Agitation (PA), Ritualistic Behaviors (RB) and Sensory Dysfunction (SD) categories, improvements were observed in 63% of volunteers. The Excessive Appetite (EA) category was the one with the lowest percentage of volunteers showing some level of improvement, just 30%, with 3% showing a moderate worsening of the symptom. Finally, there was symptomatic worsening in only five categories (Affectivity (AF), Irritability (IRR), Psychomotor Agitation (PA), Sleep Problems (SP) and Excessive appetite (EA)), with worsening being observed in no more than 10% of cases in any category.

#### 3.1.2. Parent/Caregiver-Reported Outcome Survey

**Figure 3:**
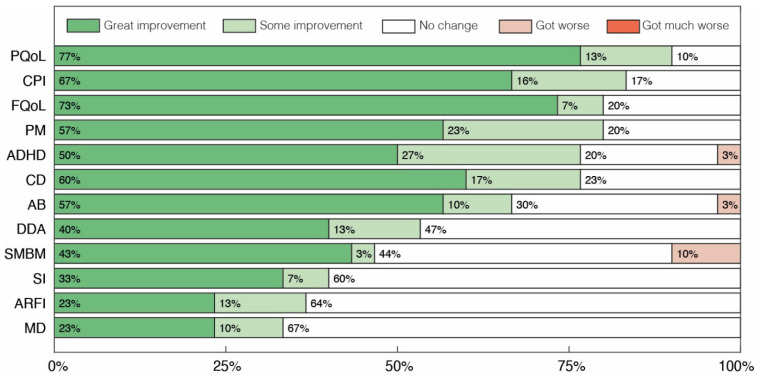
Global parents’ and caregivers’ perception of changes after treatment with cannabidiol extract. Patient’s Quality of Life (PQoL), Family’s Quality of Life (FQoL), Communication and Personal Interaction (CPI), Positive Mood States (PM), Cognitive Difficulties (CD), Attention Deficit and Hyperactivity (ADH), Abnormal Behaviors (AB), Dependence for Daily Activities (DDA), Sadness, Melancholy and Bad Mood (SMBM), Avoidance or Restriction of Food Intake (ARFI), Sleep Problems (SP) and Motor Difficulties (MD).

**Figure 4:**
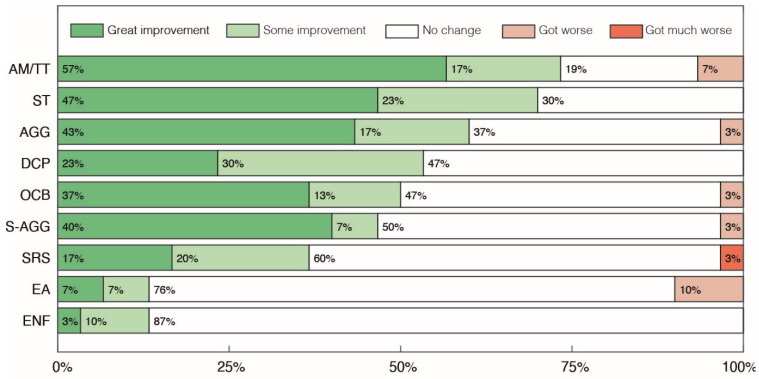
Parents’ and caregivers’ perception of changes in the sub-categories of Abnormal Behaviors after treatment with cannabidiol extract. Autistic Meltdown crisis/Temper Tantrum (AM/TT), Stereotypies (ST), Aggressiveness towards others (AGG), Discomfort in Crowded/noisy Places (DCP), Self-Aggressiveness (S-AGG), Obsessive Compulsive Behaviors (OCB), Screams and Random Sounds (SRS), Eating Non-Foods (ENF) and Excessive Appetite (EA).

**Figure 5:**
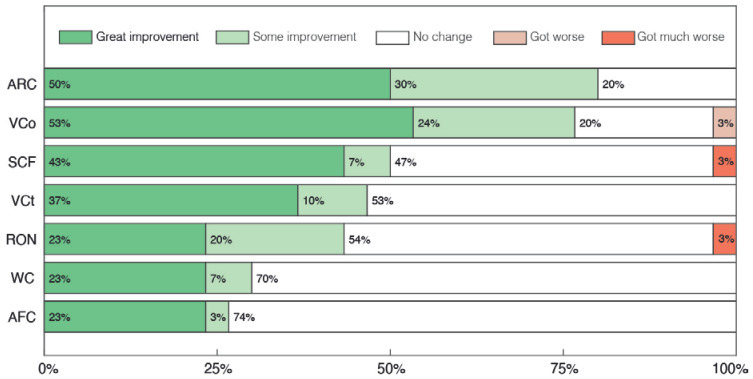
Parents’ and caregivers’ perception of changes in Communication and Social Interaction sub-categories after treatment with cannabidiol extract. Attention to Receptive direct verbal Communication (ARC), Verbal Communication (VCO), Sounds or isolated words with Communicative Functions (SCF), Visual Contact (VCT), Response to their Own Name (RON), Written Communication (WC), Alternative Forms of Communication (AFC).

The results obtained in the interviews with parents or caregivers are presented in Figure 3, Figure 4 and Figure 5. In Figure 3 we depict overall results across all categories, while Figure 4 and Figure 5 display sub-categories of two of these groups of symptoms, Abnormal Behaviors and Communication and Social Interaction, respectively. At least 33% of parents reported some level of improvement in all categories assessed (Figure 3). No case of symptomatic worsening was reported for all the general categories, and the percentage of parents who reported improvement was greater than or equal to 67% in 6 of the 12 of these categories. Amongst them, the symptomatic aspects with the highest percentage of reported improvement were Communication and Personal Interaction (CPI), Cognitive Difficulties (CD) and Attention Deficit and Hyperactivity (ADH), with great improvement of 67%, 60% and 50%, respectively. The three non-symptomatic categories evaluated also had surprising results, with at least 80% of parents reporting some level of improvement in the two quality of life sections, Patient Quality of Life (PQoL) and Family Quality of Life (FQoL), as well as in Positive Mood States (PM).

Within the sub-categories of Abnormal Behaviors, 47% of parents reported some level of improvement in 6 out of the 10 categories of symptoms assessed (Figure 4), with a higher percentage of parents reporting some improvement in the categories of Autistic Meltdown crisis/Temper Tantrum (AM/TT), Stereotypies (ST) and Aggressiveness towards others (AGG) at 74%, 70% and 60%, respectively. Worsening of symptoms was recorded in 6 out of the 10 categories, with the larger reports of worsening being found in the Excessive Appetite (EA) category, with 10% of parents reporting a moderate worsening of symptoms, and in the Autistic Meltdown crisis/Temper Tantrum (AM/TT) category, with 7% of parents reporting worsening. In the other four categories, only 3% of parents reported any type of worsening.

Regarding the sub-categories of Communication and Social Interaction, the overall perception of parents was of improvement, with at least 43% of parents reporting some improvement in 5 of the 7 categories (Figure 5). Among the categories with the greatest reports of improvement, 80% of parents reported some, moderate or great levels of improvement in the category Attention to Receptive direct verbal Communication (ARC), with 77% also reporting similar results in the category Verbal Communication (VCO). In particular, 50%, 47% and 43% of parents reported some improvement in the categories of Sounds or isolated words with Communicative Functions (SCF), Visual Contact (VCT) and Response to their Own Name (RON), respectively, which was also noticeable in the clinical evaluation in regard to the categories of Eye Contact (EC), Attention to Others (AO) and Receptive Language (RL). In the Communication and Social Interaction subsection, the categories with the lowest improvement were Written Communication (WC) and Alternative Forms of Communication (AFC), with positive change in around 30% of volunteers.

### 3.2. Results by Individual Patients

#### 3.2.1. Clinical Analysis

All individuals presented an overall positive change across clinical assessment categories. In each of the following tables, the data are presented separately for each patient, and a global Score is presented for each, indicating the average result across all categories to the left of where the Score is indicated. In Table 2, individualized clinical assessment found that all 30 volunteers obtained a positive Social Cognition Score (SCOS), with an average of 1.29. Meanwhile, the Repetitive/Restrictive and Dysfunctional Patterns Score (RDBOS) of these same volunteers was, on average, 0.85 (Table 3).

#### 3.2.2. Parent/Caregiver-Reported Outcome Survey

All volunteers’ caregivers demonstrated a general perception of improvement for every general category evaluated (Table 4). The symptom categories that presented the most improvement were Communication and Personal Interaction (CPI) and Cognitive Difficulties (CD). The category in which parents reported the lowest improvement was Avoidance or Restriction of Food Intake (ARFI). The average General Outcome Score (GOS) was 1.24.

In the sub-categories of abnormal behaviors (Table 5), Aggressiveness towards others (AGG), Self-aggressiveness (S-AGG) and Autistic Meltdown crisis/Temper Tantrum (AM/TT) had the highest average of results reported after treatment. All other categories had positive averages, the lowest being obtained for Excessive Appetite, in which three of the nine reports featured worsening of symptoms. Four volunteers had an Abnormal Behavior Score (ABOS) lower than 0. The ABOS average was 1.02 (Table 5), similar to the Communication and Social Interaction Scores (CIOS) average of 1.01 (Table 6). Out of all the Communication and Social Interaction sub-category reports (Table 6), there were three cases of worsening of symptoms and only one case of CIOS below zero.

### 3.3. Untoward Effects and Other Medication (OM)

The most commonly reported untoward effects of CBD-dominant extract treatment in this cohort (Table 7) were irritability (three cases), agitation (two cases) and aggressiveness (two cases). One case of each of the following effects was observed: fecal and urinary leakage, disobedience, stomach pain, nausea, vomiting, daytime drowsiness, insomnia, tachylalia, self-injury, intensification of binge eating and intensification of obsessive-compulsive disorder (OCD). Most of these effects were resolved or alleviated through a reduction in the dose of cannabidiol extract.

Out of the 30 volunteers enrolled in the study, 27 were taking additional medication at the onset of treatment. Of these participants, 20 volunteers either had dosage reduction and/or suspension at least one medication, amounting to 74% of participants. On the other hand, there was an increase in the dosage of some medications in four volunteers, with three other cases involving the introduction of new medication, as shown in Table 7.

## 4. Discussion

Various clinical studies, spanning retrospective and prospective approaches, have explored cannabinoids’ efficacy in alleviating ASD symptoms, with the first reports occurring in 2019. They have employed full-spectrum cannabis extracts with a broad spectrum of CBD to THC ratios [22,25,26,27,28,29,34]. However, the considerable discrepancies in sample diversity, treatment duration and outcome assessment methodologies make direct result comparison exceedingly complex.

To the best of our knowledge, this is the first study to employ a specific phytocannabinoid proportion of 33 CBD for each THC molecule. This is particularly significant given the current predominantly exploratory phase in investigating cannabis extracts for ASD treatment. Both analysis by category and by patient in our cohort demonstrated that treatment with full-spectrum cannabidiol extract was capable of generating significant global improvements in all symptomatic and non-symptomatic aspects analyzed with few untoward effects. It is important to highlight that by employing strict exclusion criteria, including only non-syndromic individuals with moderate to severe ASD, it was possible to disentangle the difference between the already known effects of cannabidiol-dominant extracts in individuals with autism and epilepsy from those observed in individuals without epilepsy. Furthermore, all individuals were previously treated for possible sleep disorders, when present, which allowed for a better differentiation of the specific improvement in this symptom category from the positive impact of better sleep on one’s mood, health and general cognitive functioning.

It is also worth noting that the evaluation of parents’ and caregivers’ perception regarding the changes caused by full-spectrum CBD oil treatment happened soon after the stipulated schedule of 6 months treatment, and the data obtained from the family were markedly consistent with the data collected in the clinical analysis, which indicates that the benefits of the treatment went beyond the clinical and medical scope and were translated and perceived by parents and caregivers, who closely monitored the day-to-day life of the individuals undergoing treatment. We believe that this correlation between the two data confirms the positive effects of the treatment from a more comprehensive perspective that considers both symptomatic and non-symptomatic aspects, complementing the clinical analysis with the daily life report and vice versa. Significantly, 74% of participants experienced either a reduction in dosage or cessation of at least one concurrent medication, underscoring the tangible therapeutic benefits of the treatment rather than any potential placebo effect or subjective bias.

Considering the results of treatment within the scope of Communication and Social Interaction, both from a clinical and family point of view, a significant positive impact was observed in aspects with a great impact on the day-to-day lives of participants, their families and their caregivers, such as eye contact, attention to others, the search for company, etc. It stands to reason that when family members and caregivers perceive the patient as more attentive and capable of establishing visual exchanges, it profoundly enhances the quality of daily interactions. At the same time, several other behaviors that permeate the interaction with that other also tend to be positively impacted, such as Communicative Intention, Affectivity and the reduction of self and hetero-aggressiveness.

Within the symptoms analyzed in the sub-categories of Abnormal Behaviors is the behavior of allotriophagia, or Pica. The term refers to the ingestion of substances or objects without nutritional value, interfering with proper gastrointestinal functioning and potentially leading to infections, injuries and poisoning [35]. This behavior is relatively common among individuals on the autism spectrum and is usually treated with applied behavior analysis (ABA) [35]; additionally, there is no drug treatment significantly effective in controlling it. In our cohort, out of the seven individuals who presented this symptom, 57% had improvement of symptoms, with no worsening in the rest of the cases. These findings are consistent with observations already made in a previous study [31], reinforcing the potential of cannabidiol extract in treating this symptom.

### Limitations

Among the inherent limitations of an observational and retrospective study, it is worth highlighting the small size of the sample group, in addition to the absence of a control group, which may end up intensifying possible placebo effects, i.e., perceptions of improvement not directly related to the treatment under study. Furthermore, due to the fact that the clinical analysis and the investigation of the family’s perception through interviews were carried out by different groups, the categories analyzed on both fronts, despite being correlated, are not exactly the same, which prevents, in many cases, a direct comparison of the two analyses.

There is also a level of subjectivity involved with data collection methods. As they are related to the perception of the medical team or family members in regard to the changes observed, there may be some optimism or pessimism bias in relation to the treatment in this assessment. In addition, some non-symptomatic categories were also evaluated which were related to more subjective aspects of the patient’s life and that of their parents and family members, as well as other more subtle behavioral aspects that may depend on the sensitivity and attention of those observing them to actually notice any changes.

## 5. Conclusions

In the present study, we show that the benefits of treatment with full-spectrum CBD oil for non-syndromic individuals with ASD are not only noticeable to the clinical eye but are also perceived and experienced by the families and caregivers. In short, the findings corroborate that this treatment, combined with a gradual and individualized dosage regimen, is safe and efficient for broader treatment of central and comorbid symptoms associated with ASD, being able to improve aspects such as social interaction, communication and quality of life.

## Figures and Tables

**Table 1:** Cohort description and dosages of cannabidiol extract at the beginning and end of treatment.

					Initial			Final				
#Patient	ASD Level	Total IQ	Age at Start of Treatment (Years)	Duration of TREATMENT (Months)	Weight (kg)	CBD (mg/day)	THC (mg/day)	Weight (kg)	CBD (mg/kg/day)	CBD (mg/day)	THC (mg/kg/day)	THC (mg/day)
1 ^m^	3/2	60 ^@^	16	6.2	55.60	55.60	1.67	56.50	2.10	118.65	0.06	3.56
2 ^f^	3/2	56 ^@^	10	7.1	61.00	61.00	1.83	62.50	1.80	112.50	0.05	3.38
3 ^f^	3/2	55 ^&^	12	6.0	57.00	57.00	1.71	58.50	4.02	235.17	0.12	7.06
4 ^m^	3/2	64 ^@^	6	6.2	26.70	26.70	0.80	27.00	4.60	124.20	0.14	3.73
5 ^m^	3/2	40 ^@^	12	6.3	48.00	48.00	1.44	50.00	4.00	200.00	0.12	6.00
6 ^m^	2/3	59 ^&^	8	6.6	38.80	38.80	1.16	38.70	2.06	79.72	0.06	2.39
7 ^m^	3	43 ^@^	17	6.2	54.00	54.00	1.62	55.00	3.27	179.85	0.10	5.40
8 ^m^	2	69 ^&^	14	6.2	53.90	53.90	1.62	52.50	3.40	178.50	0.10	5.36
9 ^m^	2/3	73 ^@^	10	7.1	63.80	63.80	1.91	64.10	2.50	160.25	0.08	4.81
10 ^m^	2	$	5	6.7	17.70	17.70	0.53	18.00	3.30	59.40	0.10	1.78
11 ^m^	3	68 ^&^	14	7.1	79.70	79.70	2.39	80.80	3.00	242.40	0.09	7.27
12 ^m^	3/2	74 ^@^	9	6.9	55.70	55.70	1.67	55.50	4.30	238.65	0.13	7.16
13 ^m^	3	$	5	6.4	19.00	19.00	0.57	21.50	5.50	118.25	0.17	3.55
14 ^f^ *	2	76 ^@^	9	6.9	26.40	26.40	0.79	30.80	4.60	141.68	0.14	4.25
15 ^m^	3	40 ^@^	10	6.9	56.10	56.10	1.68	52.50	1.93	101.33	0.06	3.04
16 ^m^	3/2	43 ^@^	14	6.2	66.30	66.30	1.99	70.20	1.70	119.34	0.05	3.58
17 ^m^	3/2	45 ^@^	9	6.2	38.50	38.50	1.16	36.50	5.30	193.45	0.16	5.80
18 ^f^ *	3	$	5	6.5	23.30	23.30	0.70	25.70	3.30	84.81	0.10	2.54
19 ^m^ *	3	40 ^@^	9	6.2	28.30	28.30	0.85	28.50	4.21	119.99	0.13	3.60
20 ^f^	3/2	65 ^@^	10	8.1	26.50	26.50	0.80	30.60	4.57	139.84	0.14	4.20
21 ^m^	2	74 ^&^	10	6.7	39.20	39.20	1.18	40.40	3.50	141.40	0.11	4.24
22 ^m^	3/2	61 ^@^	18	8.9	47.00	47.00	1.41	50.20	1.99	99.90	0.06	3.00
23 ^m^	3/2	64 ^@^	18	8.9	51.60	51.60	1.55	55.90	2.14	119.63	0.06	3.59
24 ^m^ *	3	$	7	6.5	26.20	26.20	0.79	28.4	3.00	85.2	0.09	2.56
25 ^m^	2	81 ^&^	12	3.0	37.00	37.00	1.11	39.00	4.00	156.00	0.12	4.68
26 ^m^	3	41 ^@^	18	9.7	57.00	57.00	1.71	57.00	2.20	125.40	0.07	3.76
27 ^m^	2/3	59 ^&^	14	6.0	72.70	72.70	2.18	71.40	2.25	160.65	0.07	4.82
28 ^f^	3/2	72 ^@^	13	3.9	41.00	41.00	1.23	44.00	1.50	66.00	0.05	1.98
29 ^m^	2/3	60 ^@^	15	7.6	59.60	59.60	1.79	59.00	2.10	123.90	0.06	3.72
30 ^m^	3	73 ^@^	8	6.4	29.00	29.00	0.87	28.00	1.20	33.60	0.04	1.01
Mean		59.8	11.23	6.6	45.22	45.22	1.36	46.91	3.11	135.32	0.09	4.06

* Participants who had previously used pure CBD before treatment. Starting doses for all participants were 1 mg/kg/day of CBD (0.03 mg/kg/day of THC). ^m^ = male, ^f^ = female, ^&^ = WISC, ^@^ = WASI, $ = SON-R.

**Table 2:** Clinical assessment of individuals in social cognition after treatment with cannabidiol extract.

#Patient	EC	AO	CS	AF	CI	EL	RL	LE	DRP	DLA	SCOS
1 ^m^	1	2	1	2	1	0	0	1	1	1	1
2 ^f^	1	1	1	2	1	1	1	2	1	1	1.2
3 ^f^	1	2	1	1	1	1	2	2	1	2	1.4
4 ^m^	2	1	2	2	2	2	1	1	2	2	1.7
5 ^m^	1	1	1	2	2	2	2	1	2	2	1.6
6 ^m^	2	2	1	2	2	2	2	2	2	2	1.9
7 ^m^	2	2	2	2	2	2	2	1	0	2	1.7
8 ^m^	NaN	1	NaN	2	2	2	2	2	NaN	2	1.9
9 ^m^	2	2	1	1	2	1	1	0	1	0	1.1
10 ^m^	2	2	2	2	2	2	2	2	2	2	2
11 ^m^	1	0	1	1	1	2	1	2	0	0	0.9
12 ^m^	1	1	1	2	1	2	2	2	1	2	1.5
13 ^m^	2	2	2	2	2	2	2	2	2	2	2
14 ^f^ *	1	2	2	1	2	0	1	1	1	2	1.3
15 ^m^	1	2	1	0	1	0	1	1	1	2	1.1
16 ^m^	1	1	NaN	1	1	2	2	2	2	2	1.6
17 ^m^	2	1	2	2	2	2	2	2	1	2	1.8
18 ^f^ *	2	1	1	2	2	0	2	1	1	0	1.3
19 ^m^ *	0	1	0	1	1	0	1	2	1	0	0.8
20 ^f^	2	2	2	1	2	2	1	1	1	1	1.5
21 ^m^	NaN	1	NaN	1	1	2	2	2	1	2	1.5
22 ^m^	0	0	1	1	0	0	0	1	1	1	0.5
23 ^m^	2	1	2	1	1	2	1	2	2	2	1.6
24 ^m^ *	1	1	2	0	1	0	1	1	0	0	0.7
25 ^m^	0	1	1	−1	1	2	0	2	1	0	0.7
26 ^m^	0	1	2	0	2	1	1	0	0	0	0.7
27 ^m^	NaN	1	NaN	1	NaN	0	0	0	0	0	0.3
28 ^f^	2	2	2	2	2	2	2	1	1	2	1.8
29 ^m^	1	1	1	1	NaN	2	1	1	0	1	1
30 ^m^	1	0	0	0	1	0	0	1	2	1	0.6
n	27	30	26	30	28	30	30	30	29	30	
Mean	1.26	1.27	1.35	1.23	1.46	1.27	1.27	1.37	1.07	1.27	1.29

^m^ = male, ^f^ = female, * Participants who had previously used pure CBD before treatment. Eye contact (EC), Attention to Others (AO), Company seeking (CS), Affectivity (AF), Communicative Intent (CI), Expressive Language (EL), Receptive Language (RL), Learning (LE), Dysfunctional/Repetitive Play (DRP), Daily Life Activities (DLA) and SCOS: Social Cognition Outcome Score. The numbers denote 2—great improvement, 1—some improvement, 0—no change, −1—some worsening, −2—great worsening. Numbers are color-coded in a pseudo-heat map similar to the colors used in the figures.

**Table 3:** Clinical assessment of individuals in repetitive/restrictive and dysfunctional behavior after treatment with cannabidiol extract.

#Patient	S-AGG	AGG	IRR	PA	MST	VST	ECH	RB	SD	SP	EA	ARFI	RDBOS
1 ^m^	2	2	2	0	NaN	NaN	2	1	1	NaN	NaN	0	1.1
2 ^f^	2	2	2	2	1	0	2	1	1	NaN	2	NaN	1.3
3 ^f^	NaN	NaN	0	NaN	0	2	2	1	2	NaN	NaN	2	1
4 ^m^	2	2	2	2	1	NaN	NaN	1	2	2	NaN	2	1.7
5 ^m^	2	2	2	1	0	2	NaN	1	1	2	1	NaN	1
6 ^m^	2	2	2	1	0	2	NaN	1	0	NaN	2	NaN	0.8
7 ^m^	NaN	2	2	2	NaN	NaN	NaN	1	1	NaN	NaN	NaN	1.3
8 ^m^	NaN	NaN	NaN	2	0	NaN	NaN	1	0	NaN	NaN	NaN	0.3
9 ^m^	2	2	2	1	0	0	0	0	1	−2	2	1	0.5
10 ^m^	1	2	2	2	1	0	1	1	2	NaN	NaN	2	1
11 ^m^	NaN	2	2	NaN	0	2	2	0	1	2	2	NaN	1
12 ^m^	2	2	2	2	0	0	NaN	1	0	2	1	NaN	0.9
13 ^m^	2	2	2	2	0	2	2	2	2	2	NaN	2	1.3
14 ^f^ *	NaN	2	2	2	1	NaN	2	1	2	2	NaN	1	1.5
15 ^m^	2	2	2	2	0	0	NaN	0	2	NaN	2	2	1.4
16 ^m^	NaN	2	2	2	2	2	2	0	0	1	1	NaN	1
17 ^m^	NaN	NaN	2	1	1	2	2	1	2	NaN	2	2	1.5
18 ^f^ *	2	2	2	2	1	2	2	1	2	1	NaN	1	1.5
19 ^m^ *	1	2	1	1	0	0	0	1	1	−1	NaN	1	0.5
20 ^f^	2	2	2	2	2	NaN	2	1	2	2	NaN	2	1.9
21 ^m^	1	1	2	1	0	0	0	0	2	2	NaN	1	0.8
22 ^m^	2	2	0	NaN	0	NaN	NaN	1	1	2	NaN	0	0.7
23 ^m^	2	NaN	1	NaN	0	NaN	NaN	2	1	2	NaN	2	1.2
24 ^m^ *	NaN	1	1	1	1	1	0	0	0	NaN	NaN	NaN	0.6
25 ^m^	NaN	NaN	−2	0	NaN	NaN	NaN	0	0	0	NaN	0	−0.4
26 ^m^	NaN	NaN	0	0	0	1	1	1	0	NaN	NaN	NaN	0.4
27 ^m^	NaN	NaN	−2	−2	0	NaN	0	0	0	NaN	−1	NaN	−0.7
28 ^f^	NaN	1	0	0	0	0	1	0	0	NaN	NaN	2	0.4
29 ^m^	NaN	0	−1	−2	1	1	1	0	0	NaN	NaN	NaN	0
30 ^m^	0	0	0	−1	0	0	0	0	0	0	0	NaN	0
n	17	23	29	26	27	20	20	30	29	16	10	17	
Mean	1.71	1.7	1.17	1	0.44	0.95	1.2	0.7	1	1.19	1.2	1.35	0.85

^m^ = male, ^f^ = female, * Participants who had previously used pure CBD before treatment. Self-aggressiveness (S-AGG), Aggressiveness towards others (AGG), Irritability (IRR), Psychomotor Agitation (PA), Motor Stereotypies (MST), Vocal Stereotypies (VST), Echolalias (ECH), Ritualistic Behaviors (RB), Sensory Dysfunction (SD), Sleep Problems (SP), Excessive appetite (EA), Avoidance and Restriction of Food Intake (ARFI) and RDBOS: Repetitive/restrictive and Dysfunctional Behaviors Outcome Score. The numbers denote 2—great improvement, 1—some improvement, 0—no change, −1—some worsening, −2—great worsening. Numbers are color-coded in a pseudo-heat map similar to the colors used in the figures.

**Table 4:** Parents’ and caregivers’ individual perceptions of changes in the general symptomatic and non-symptomatic categories after treatment with cannabidiol extract.

#Patient	ADH	AB	SMBM	MD	DDA	CPI	CD	SP	ARFI	GOS	PM	PQoL	FQoL
1 ^m^	0	0	NaN	2	NaN	2	NaN	2	1	1.2	2	2	1
2 ^f^	1	2	NaN	NaN	2	2	2	NaN	1	1.7	1	2	2
3 ^f^	1	0	2	NaN	1	1	2	NaN	0	1	1	2	2
4 ^m^	2	2	NaN	NaN	1	2	1	NaN	0	1.3	0	2	2
5 ^m^	2	2	2	2	2	2	2	2	1	1.9	2	2	2
6 ^m^	2	2	2	0	0	2	2	NaN	0	1.3	1	2	2
7 ^m^	2	NaN	2	2	2	2	2	1	NaN	1.9	2	2	2
8 ^m^	2	2	2	2	2	2	2	NaN	2	2	2	2	2
9 ^m^	2	2	1	1	2	2	2	1	2	1.7	2	2	2
10 ^m^	NaN	2	0	0	2	2	1	0	1	1	0	2	2
11 ^m^	1	2	2	0	NaN	2	2	0	NaN	1.3	2	2	2
12 ^m^	1	2	2	NaN	NaN	2	2	NaN	0	1.5	2	2	2
13 ^m^	0	2	2	2	2	2	2	NaN	2	1.8	2	2	2
14 ^f^	2	2	2	1	0	1	1	2	0	1.2	1	2	2
15 ^m^	2	2	0	NaN	0	2	1	2	0	1.1	2	2	2
16 ^m^	2	2	2	2	2	2	2	2	NaN	2	2	2	2
17 ^m^	1	2	0	NaN	2	2	2	NaN	0	1.3	2	2	2
18 ^f^	2	0	NaN	NaN	2	2	NaN	0	0	1	2	2	2
19 ^m^	2	2	NaN	NaN	NaN	2	2	NaN	0	1.6	2	2	2
20 ^f^	2	NaN	2	1	NaN	2	2	2	2	1.9	2	2	2
21 ^m^	2	2	2	2	1	2	2	2	2	1.9	1	2	2
22 ^m^	NaN	1	NaN	NaN	2	1	2	2	2	1.7	2	2	2
23 ^m^	NaN	2	2	NaN	2	2	2	NaN	2	2	2	2	2
24 ^m^ *	1	0	−1	NaN	1	0	2	0	NaN	0.4	1	0	0
25 ^m^	0	0	0	NaN	0	0	0	0	0	0	0	0	0
26 ^m^	2	0	NaN	NaN	NaN	0	0	NaN	NaN	0.5	0	1	0
27 ^m^	2	0	−1	0	0	1	0	2	NaN	0.5	1	1	1
28 ^f^	−1	−1	−1	NaN	NaN	0	0	NaN	0	−0.5	0	0	0
29 ^m^	1	1	0	0	0	1	1	0	0	0.4	2	1	0
30 ^m^	1	1	0	0	0	0	0	2	NaN	0.5	NaN	1	0
n	27	28	23	16	23	30	28	18	23	--	29	30	30
Mean	1.37	1.29	1.04	1.06	1.22	1.5	1.46	1.22	0.78	1.24	1.41	1.67	1.53

^m^ = male, ^f^ = female, * Participants who had previously used pure CBD before treatment. Attention Deficit and Hyperactivity (ADH), Abnormal Behaviors (AB), Sadness, Melancholy and Bad Mood (SMBM), Motor Difficulties (MD), Dependence for Daily Activities (DDA), Communication and Personal Interaction (CPI), Cognitive Difficulties (CD), Sleep Problems (SP), Avoidance or Restriction of Food Intake (ARFI), GOS: General Outcome Score, Positive Mood States (PM), Patient’s Quality of Life (PQoL) and Family’s Quality of Life (FQoL). The numbers denote 2—great improvement, 1—some improvement, 0—no change, −1—some worsening, −2—great worsening. Numbers are color-coded in a pseudo-heat map similar to the colors used in the figures.

**Table 5:** Parents’ and caregivers’ individual perceptions of changes in the sub-categories of Abnormal Behaviors after treatment with cannabidiol extract.

#Patient	ST	AGG	S-AGG	AM/TT	SRS	OCB	ENF	DCP	EA	ABOS
1 ^m^	2	2	2	2	NaN	1	NaN	1	0	1.5
2 ^f^	0	0	0	2	0	0	1	1	NaN	0.4
3 ^f^	NaN	2	NaN	2	0	0	NaN	1	NaN	0.8
4 ^m^	2	2	2	2	NaN	NaN	1	2	NaN	1.9
5 ^m^	2	2	2	1	2	2	NaN	1	0	1.6
6 ^m^	1	NaN	NaN	1	2	2	0	0	NaN	0.9
7 ^m^	2	2	NaN	2	NaN	2	NaN	2	1	1.9
8 ^m^	2	2	2	2	2	2	NaN	0	2	1.6
9 ^m^	1	2	NaN	NaN	1	0	NaN	0	NaN	1
10 ^m^	2	2	2	2	NaN	2	NaN	2	NaN	2
11 ^m^	NaN	NaN	2	1	NaN	NaN	NaN	NaN	−1	1
12 ^m^	1	NaN	2	NaN	NaN	2	NaN	0	NaN	1.2
13 ^m^	2	NaN	2	NaN	NaN	2	NaN	2	NaN	2
14 ^f^	0	2	2	2	NaN	2	NaN	NaN	2	1.4
15 ^m^	1	2	NaN	2	1	NaN	NaN	2	0	1.4
16 ^m^	0	NaN	NaN	0	NaN	0	NaN	0	−1	−0.2
17 ^m^	2	1	NaN	2	1	2	2	0	NaN	1.2
18 ^f^	2	2	2	2	2	1	NaN	2	0	1.7
19 ^m^	2	1	1	2	1	NaN	0	NaN	NaN	1
20 ^f^	1	2	NaN	2	NaN	2	NaN	1	−1	1
21 ^m^	2	2	2	2	2	2	1	2	NaN	1.9
22 ^m^	1	1	1	1	−2	0	NaN	1	0	0.4
23 ^m^	2	1	2	2	0	0	0	1	0	1
24 ^m^ *	0	−1	−1	−1	0	NaN	0	NaN	NaN	−0.5
25 ^m^	NaN	NaN	NaN	−1	NaN	NaN	NaN	0	NaN	−0.5
26 ^m^	0	NaN	NaN	2	1	0	0	1	0	0.7
27 ^m^	1	NaN	NaN	1	0	1	NaN	0	0	0.6
28 ^f^	NaN	NaN	NaN	0	NaN	−1	0	0	NaN	−0.3
29 ^m^	2	NaN	NaN	2	NaN	1	NaN	1	1	1.5
30 ^m^	2	1	0	0	1	0	0	NaN	0	0.5
n	26	20	17	27	17	24	11	25	16	--
Mean	1.35	1.5	1.47	1.37	0.82	1.04	0.45	0.92	0.19	1.02

^m^ = male, ^f^ = female, * Participants who had previously used pure CBD before treatment. Stereotypies (ST), Aggressiveness towards others (AGG), Self-aggressiveness (S-AGG), Autistic Meltdown crisis/Temper Tantrum (AM/TT), Screams and Random Sounds (SRS), Obsessive Compulsive Behaviors (OCB), Eating Non-Foods (ENF), Discomfort in Crowded/noisy Places (DCP), Excessive Appetite (EA) and ABOS: Abnormal Behaviors Outcome Score. The numbers denote 2—great improvement, 1—some improvement, 0—no change, −1—some worsening, −2—great worsening. Numbers are color-coded in a pseudo-heat map similar to the colors used in the figures.

**Table 6:** Parents’ and caregivers’ individual perceptions of changes in Communication and Social Interaction sub-categories after treatment with cannabidiol extract.

#Patient	VCO	VCT	RON	ARC	SCF	WC	AFC	CIOS
1 ^m^	2	1	0	1	0	0	0	0.6
2 ^f^	2	1	1	2	0	2	2	1.4
3 ^f^	1	NaN	2	2	1	NaN	2	1.6
4 ^m^	1	2	2	2	2	2	NaN	1.8
5 ^m^	2	2	2	2	2	2	2	2
6 ^m^	0	2	2	2	0	NaN	2	1.3
7 ^m^	1	2	0	2	0	NaN	2	1.2
8 ^m^	1	2	2	1	2	NaN	NaN	1.6
9 ^m^	2	2	1	2	2	2	1	1.7
10 ^m^	NaN	NaN	0	2	2	2	NaN	1.5
11 ^m^	2	NaN	−2	2	0	NaN	NaN	0.5
12 ^m^	1	NaN	1	1	2	0	NaN	1
13 ^m^	2	2	2	2	−2	NaN	NaN	1.2
14 ^f^	2	0	0	1	0	0	NaN	0.5
15 ^m^	2	0	1	2	2	2	NaN	1.5
16 ^m^	2	2	0	2	2	1	NaN	1.5
17 ^m^	2	1	0	1	2	1	NaN	1.2
18 ^f^	2	0	0	1	2	2	NaN	1.2
19 ^m^	0	2	2	2	0	NaN	2	1.3
20 ^f^	1	0	0	2	0	NaN	NaN	0.6
21 ^m^	2	2	1	2	2	NaN	2	1.8
22 ^m^	2	2	0	0	2	NaN	0	1
23 ^m^	2	0	0	1	2	NaN	NaN	1
24 ^m^ *	0	NaN	1	1	1	NaN	0	0.6
25 ^m^	2	0	0	0	0	0	NaN	0.3
26 ^m^	0	0	0	0	0	NaN	NaN	0
27 ^m^	−1	0	0	0	0	0	NaN	−0.2
28 ^f^	1	0	0	0	0	0	NaN	0.2
29 ^m^	2	0	0	1	0	0	NaN	0.5
30 ^m^	0	0	0	0	0	0	NaN	0
n	29	25	30	30	30	17	11	-
Mean	1.31	1	0.6	1.3	0.87	0.94	1.36	1.01

^m^ = male, ^f^ = female, * Participants who had previously used pure CBD before treatment. Verbal Communication (VCO), Visual Contact (VCT), Response to their Own Name (RON), Attention to Receptive direct verbal Communication (ARC), Sounds or isolated words with Communicative Functions (SCF), Written Communication (WCO), Alternative Forms of Communication (AFC) and CIOS: Communication and Interaction Outcome Score. The numbers denote 2—great improvement, 1—some improvement, 0—no change, −1—some worsening, −2—great worsening. Numbers are color-coded in a pseudo-heat map similar to the colors used in the figures.

**Table 7:** Other medication (OM) used by volunteers and untoward effects observed during treatment with cannabidiol extract.

#Patient	Other Medication at Treatment Onset	Other Medication at the End of Treatment	Summary of Alterations in Other Medication	Untoward Effects
1 ^m^	Risperidone	Risperidone	Increased dosage	Aggressiveness
2 ^f^	Fluoxetine and Acetazolamide	Fluoxetine	Suspension of Acetazolamide	N
3 ^f^	N	N	N	N
4 ^m^	Haloperidol	Haloperidol	Reduced dosage	N
5 ^m^	Fluoxetine and Periciazine	Fluoxetine and Periciazine	Reduced dosage of all OM	N
6 ^m^	Aripiprazole	Aripiprazole	N	N
7 ^m^	Periciazine	Periciazine	Increased dosage	N
8 ^m^	Methylphenidate and Fluoxetine	Methylphenidate	Suspension of Fluoxetine	N
9 ^m^	Risperidone and Citalopram	Risperidone, Citalopram and Levomepromazine	Reduced dosage of Citalopram; Introduction of Levomepromazine	N
10 ^m^	Melatonin e Risperidone	Risperidone	Reduced dosage of Risperidone; Suspension of Melatonin.	Nausea and Vomiting
11 ^m^	Periciazine	Periciazine	N	N
12 ^m^	Levomepromazine and Carbamazepine	Levomepromazine and Carbamazepine	Reduced dosage of Levomepromazine	N
13 ^m^	Risperidone, Melatonin and Levomepromazine	Risperidone, Melatonin	Reduced dosage of Risperidone; Suspension of Levomepromazine	N
14 ^f^ *	Risperidone	Risperidone	N	N
15 ^m^	Risperidone e Carbamazepine	Risperidone e Carbamazepine	Reduced dosage of Risperidone	N
16 ^m^	Methylphenidate and Fluoxetine	Fluoxetine	Suspension of Methylphenidate	N
17 ^m^	Risperidone	Risperidone	N	N
18 ^f^ *	Melatonin, CBD	Melatonin	Suspension do CBD	N
19 ^m^ *	Haloperidol	Levomepromazine	Suspension of Haloperidol. Introduction of Levomepromazine	Irritability and Insomnia
20 ^f^	Risperidone and Methylphenidate	Risperidone	Suspension of Methylphenidate; Increased dosage of Risperidone	Aggressiveness, Irritability, Self-injury
21 ^m^	Levetiracetam and Risperidone	Risperidone	Suspension of Levetiracetam; Reduced Dosage of Risperidone	N
22 ^m^	Fluoxetine and Risperidone	Fluoxetine	Reduced dosage of Fluoxetine; Suspension of Risperidone	N
23 ^m^	Fluoxetine and Risperidone	N	Suspension of Fluoxetine and Risperidone	N
24 ^m^ *	Pure CBD, Melatonin, Aripiprazole and Periciazine	Melatonin, Aripiprazole Periciazine	Suspension of Pure CBD; Reduced dosage of Aripiprazole and Melatonin	Fecal and Urinary Leaks
25 ^m^	Methylphenidate and Carbamazepine	Methylphenidate	Suspension of Carbamazepine	Disobedience, Irritability
26 ^m^	N	N	N	Stomach Pain
27 ^m^	Citalopram and Risperidone	Risperidone and Sertraline	Suspension of Citalopram; Introduction of Sertraline	Intensification of Binge Eating
28 ^f^	Fluvoxamine and Risperidone	Fluvoxamine and Risperidone	Increased dosage of Fluvoxamine	Intensification of OCD, Agitation, Daytime Drowsiness
29 ^m^	Risperidone	Risperidone	Reduced dosage	Agitation, Taquilalia
30 ^m^	N	N	N	N

* Participants who had already used pure CBD before treatment. ^m^ = male, ^f^ = female, N = None.

## Data Availability

Data is contained within the article and Supplementary Material.

## Supplementary Materials

The following supporting information can be downloaded at: https://www.mdpi.com/article/10.3390/ph17060686/s1, Table S1: Height, Weight, Body Mass Index (BMI) and BMI variation (final-initial) for all participants.
