# Supplementary material for: Clinical and Family Implications of Cannabidiol (CBD)-Dominant Full-Spectrum Phytocannabinoid Extract in Children and Adolescents with Moderate to Severe Non-Syndromic Autism Spectrum Disorder (ASD): An Observational Study on Neurobehavioral Management
Source: Pharmaceuticals (Basel). 2024 May 27;17(6):686. doi: 10.3390/ph17060686 (PMC11206937; doi:10.3390/ph17060686)
Supplement: Supplementary file 1 [file pharmaceuticals-17-00686-s001.zip › pharmaceuticals-2972983-supplementary.pdf]

Supplementary Table S1. Height, Weight, Body Mass Index (BMI) and BMI variation (final-initial) for all participants.

	Initial			Final			
#Pacient	Height (m)	Weight (kg)	BMI	Height (m)	Weight (kg)	BMI	BMI variation
1m	1.8	55.6	17.2	1.81	56.5	17.3	0.2
2f	1.48	61	27.8	1.51	62.5	27.4	-0.4
3f	1.56	57	23.4	1.56	58.5	24,0	0.6
4m	1.27	26.7	16.6	1.34	27	15.1	-1.4
5m	1.59	48	19,0	1.65	50	18.4	-0.6
6m	1.34	38.8	21.8	1.36	38.7	21.1	-0.7
7m	1.76	54	17.4	1.76	55	17.8	0.3
8m	1.6	53.9	21.1	1.61	52.5	20.3	-0.8
9m	1.5	63.8	28.5	1.5	64.1	28.5	-0.1
10m	1.05	17.7	16.1	1.08	18	15.4	-0.6
11m	1.72	79.7	27.1	1.78	80.8	25.5	-1.6
12m	1.39	55.7	28.8	1.41	55.5	28.1	-0.7
13m	1.14	19	14.7	1.17	21.5	15.7	1,0
14f*	1.38	26.4	13.9	1.4	30.8	15.7	1.9
15m	1.42	56.1	27.8	1.49	52.5	23.8	-4,0
16m	1.55	66.3	27.6	1.57	70.2	28.5	0.9
17m	1.38	38.5	20.2	1.4	36.5	18.6	-1.6
18f*	1.17	23.3	17,0	#	25.7	#	#
19m*	#	28.3	#	#	28.5	#	#
20f	1.41	26.5	13.4	1.49	30.6	13.8	0.4
21m	1.37	39.2	20.8	1.41	40.4	20.3	-0.4
22m	1.71	47	16.2	1.71	50.2	17.3	1.1
23m	1.72	51.6	17.4	1.72	55.9	18.9	1.5
24m*	1.28	26.2	16,0	#	28.4	#	#
25m	1.56	37	15.2	1.6	39	15.2	0,0
26m	1.74	57	18.8	1.76	57	18.5	-0.3
27m	1.63	72.7	27.4	1.65	71.4	26.2	-1.1
28f	1.58	41	16.4	1.59	44	17.4	1,0
29m	1.73	59.6	19.9	1.8	59	18.2	-1.7
30m	1.31	29	16.9	1.37	28	14.9	-2,0
**Mean**	**1.49**	**45.22**	**20.2**	**1.54**	**46.91**	**20.1**	**-0.35**

*Participants who had previously used pure CBD before treatment. # Data not available due to technical problems during clinical assessment.
